# A *Sox2* BAC Transgenic Approach for Targeting Adult Neural Stem Cells

**DOI:** 10.1371/journal.pone.0049038

**Published:** 2012-11-07

**Authors:** Wenfei Kang, Jean M. Hébert

**Affiliations:** Departments of Neuroscience and Genetics, Albert Einstein College of Medicine, Bronx, New York, United States of America; Baylor College of Medicine, United States of America

## Abstract

The transcription factor gene *Sox2* is expressed in embryonic neural stem/progenitor cells and previous evidence suggests that it is also expressed in adult neural stem cells. To target *Sox2*-expressing neural stem/progenitor cells in a temporal manner, we generated a bacterial artificial chromosome (BAC) transgenic mouse line, in which an inducible form of Cre, CreER™, is expressed under *Sox2* regulatory elements. Inducible Cre activity in these mice was characterized using floxed reporters. During development, the *Sox2-CreER* transgenic mice show inducible Cre activity specifically in CNS stem/progenitor cells, making them a useful tool to regulate the expression of floxed genes temporally in embryonic neural stem/progenitor cells. In the adult, we examined the cell-specific expression of *Sox2* and performed long-term lineage tracing. Four months after the transient induction of Cre activity, recombined GFAP+ stem-like cells and DCX+ neuroblasts were still abundant in the neurogenic regions including the subventricular zone (SVZ), rostral migratory stream (RMS), and subgranular zone (SGZ) of the dentate gyrus. These results provide definitive in vivo evidence that *Sox2* is expressed in neural stem cells (NSC) in both the SVZ and SGZ that are capable of self-renewal and long-term neurogenesis. Therefore, *Sox2-CreER* mice should be useful in targeting floxed genes in adult neural stem cells.

## Introduction

Evidence suggests that the HMG (high-mobility-group) box transcription factor gene *Sox2* marks neural stem/progenitor cells throughout development and in the adult [1], [2]. Studies using *Sox2-*β*geo* or *Sox2-GFP* knock-in mice and staining with anti-SOX2 antibodies have found that *Sox2* expression is restricted to the proliferating neural progenitor cell population during central nervous system (CNS) development, is down-regulated in differentiating cells, and is absent in cells that have exited the cell cycle and acquired a neuronal identity in the developing cortex [3], [4], [5], [6].

Consistent with its expression pattern, *Sox2* plays an important role in early neural development in Xenopus, Drosophila, chick, and mouse [2], [7], [8]. In chick, constitutive expression of *SOX2* inhibits neuronal differentiation and maintains neural progenitor identity, whereas inhibition of *SOX2* promotes cell cycle exit of progenitors and early neuronal differentiation [9]. Due to its essential requirement in epiblast development, loss-of-function *Sox2* mouse mutants fail to survive after implantation. However, in *Sox2* hypomorphic mice and in neural-specific knockout mutants of *Sox2* during development, loss of GFAP+ neural stem cells (NSC), precursor cells, and neurogenesis were observed in mature neurogenic regions including the dentate gyrus of the hippocampus and the subventricular zone (SVZ) [6], [10]. These data indicate that *Sox2* is required for the maintenance of neural stem/precursor cells during development.

Similar to its expression pattern and function during development, in adult mice, *Sox2* is expressed in radial glial-like stem cells marked by GFAP and in proliferating precursor cells in the two neurogenic regions, the SVZ and subgranular zone (SGZ) of the dentate gyrus. In culture, *Sox2*-expressing cells from adult neurogenic regions exhibit self-renewing and multipotent NSC properties, suggesting that *Sox2*-expressing cells include NSCs [3], [11]. Conditional deletion of *Sox2* in adult mice reduces the number of GFAP/nestin+ radial glia stem cells and the proliferation of precursor cells in the hippocampus, indicating that *Sox2* is required for NSC maintenance in the adult hippocampus [10].

In addition to its expression in neural stem/precursor cells in the adult brain, SOX2 protein was found in many mature astrocytes and in rare differentiated neurons in the neocortex and striatum and abundantly in the thalamus [6], [12], [13]. However, in the *Sox2-GFP* knock-in mice, GFP+ cells do not co-label with differentiated neuronal markers and only occasionally with the astrocytic marker GFAP in the corpus callosum [11], casting doubt on whether *Sox2* is expressed or not in mature neurons and astrocytes outside the corpus callosum. Functionally, adult *Sox2* hypomorphic mice exhibit epileptic spikes, motor dysfunction, and neuronal degeneration in the striatum, septum, and thalamus, suggesting a possible direct role for *Sox2* in adult neuronal function and/or survival [6]. However, whether these abnormalities reflect a developmental defect rather than a direct requirement for *Sox2* in differentiated adult cell types remains unclear.

To date, the expression of *Sox2* in differentiated cells in the adult brain has not been fully characterized. Here, we used a bacterial artificial chromosome (BAC)-based *Sox2* transgenic mouse to examine the expression of *Sox2* in the adult brain. We provide in vivo evidence that *Sox2* is expressed in adult neural stem cells that are capable of self-renewal and long-term neurogenesis in both the SVZ and SGZ. Interestingly, *Sox2* is also expressed in certain neurons and mature astrocytes in several brain regions including the neocortex, hippocampus, basal ganglia, midbrain, and hindbrain, suggesting roles not only in neural stem cells, but also differentiated cell types in the adult brain.

## Materials and Methods

### Generation of *Sox2-CreER* BAC transgenic mice

The RP23-2B8 BAC contains roughly 155 Kb upstream of the transcriptional start site of *Sox2* and roughly 65 Kb downstream of the end of the coding region. The coding sequence of *Sox2* in RP23-2B8 was replaced with the coding sequence for CreER™ as described previously [14] and the modified BAC DNA was microinjected into pronuclei of fertilized oocytes from FVB mice. Founder mice that carried the transgenic BAC were identified by PCR and confirmed by Southern blot. The founder lines were crossed with Swiss-Webster mice and genotyped using the following primers to identify the presence of Cre gene: forward, 5′ GTCGAAATCAGTGCGTTCGAAC 3′; reverse, 5′ GTTCGCAAGGAACCTGATGGAC 3′. To examine the expression pattern of the transgene, heterozygous *Rosa26^lox-stop-lox-lacZ^* [15] or RCE [16] reporters were bred to hemizygous *Sox-CreER™* transgenic mice. The progeny containing one copy of the reporter allele and the *Sox2-CreER* allele were used for all experiments. Experiments with mice were carried out in accordance with the strict guidelines set by the NIH and all experiments were approved by the Einstein IACUC committee (protocol number 20110601). Perfusions were performed under general ketamine/xylazine anesthesia.

### Tamoxifen treatment

Tamoxifen (TM) was dissolved in corn oil at 20 mg/ml. TM was administered intraperitoneally at 5 mg/35 g body weight to pregnant females the day before embryos were collected or every other day for a total of 5 doses for analyses of adult brains. Brains were collected for analysis at different time points, as indicated.

### Immunofluorescence

Mice were perfused using 4% paraformaldehyde (PFA) before post-fixing overnight in 4% PFA at 4°C. Samples were then cryoprotected in 20% sucrose and embedded in OCT. Tissues were sectioned at 20 µm. Immunostaining was performed according to standard methods. Briefly, sections were incubated with primary antibodies in the blocking solution overnight at 4°C, incubated with appropriate Texas Red or FITC-conjugated secondary antibodies in the blocking solution for 1 hour at room temperature, and mounted in Fluoromount G. The following primary antibodies were used: mouse anti-NeuN (1∶100, Millipore), rabbit anti-GFAP(1∶500, Dako Cytomation), rabbit anti-S100 (1∶100, Sigma), rabbit anti-GFP (1∶100, Invitrogen), rabbit anti-GABA (1∶500, Millipore), mouse anti-Calbindin (1∶1000, Sigma), rabbit anti-Calretinin (1∶1000, Chemicon), rat anti-Somatostatin (1∶50, Millipore), rabbit anti-GFAP (1∶500, Dako Cytomation), rabbit anti-DCX (1∶1000, Millipore), mouse anti-CC1(1∶100, Calbiochem), rabbit anti-Parvalbumin (1∶5000, Swant), chicken anti-β-gal (1∶200. abcam), rabbit anti-Sox2 (1∶100, Millipore). For chicken anti-β-gal staining, a TSA kit (PerkinElmer) was used to enhance the signal after the secondary antibody. Sections were analyzed by conventional fluorescence microscopy (Zeiss AxioSkop2 p) except for GFAP/GFP and GFAP/DCX co-labeling experiments which were analyzed by confocal fluorescence microscope (Zeiss LSM 5 Duo Scanner).

### X-gal Staining

For whole mount staining, embryos at E10.5 were dissected and fixed in 1% formaldehyde, 0.2% glutaraldehyde, 2 mM MgCl_2_, 5 mM EGTA, 0.02% NP-40 for 1 hour at 4°C. After several washes in PBS, they were stained at 37°C in the dark in 5 mM K_3_Fe(CN)_6_, 5 mM K_4_Fe(CN)_6_, 2 mM MgCl_2_, 0.01% deoxycholic acid, 0.02% NP-40, and 1 mg/ml X-gal. For sections, after dissection, embryos are fixed with 4% PFA in PBS at 4°C for 1 hour, equilibrated in 30% sucrose/PBS and embedded in OCT. 40 µm cryosections were subjected to staining as described above for whole mount staining. After several washes in PBS, sections were counterstained with neutral red.

## Results and Discussion

### The generation of *Sox2-CreER* transgenic mice

**Figure 1:**
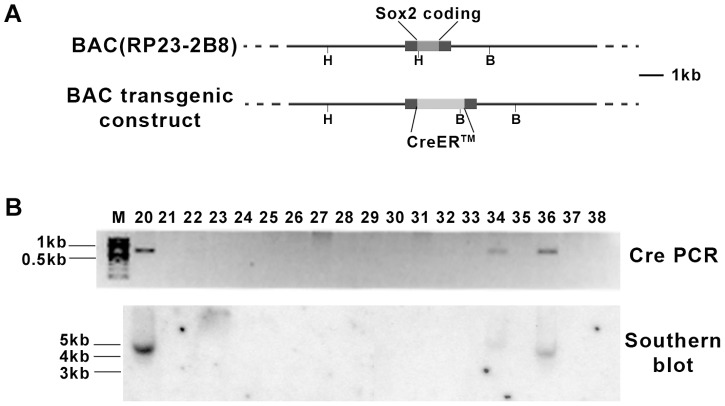
Generation of *Sox2-CreER* BAC transgenic mice. (A) Illustration of the transgene construction. In the BAC clone RP23-2B8, the *Sox2* coding sequence was replaced by CreER™ sequence. The two black boxes represent the 5′UTR and 3″ UTR of the *Sox2* gene. H, HindIII; B, BglI. (B) Positive founder lines (No.20, 34, and 36) were identified by PCR and Southern blot. For PCR, Cre sequence was amplified. For Southern blot, genomic DNA was digested with HindIII and BglI and probed with a Cre sequence.

To investigate the expression pattern of *Sox2* in specific cell types in the adult brain and to develop a tool for manipulating gene expression in *Sox2*-expressing cells, we generated a tamoxifen (TM)-inducible transgenic line to express the Cre DNA recombinase in *Sox2*-expressing cells. Since the cis-regulatory sequences of *Sox2* in mammals has not been completely defined [5], we used a ∼200 Kb *Sox2* bacterial artificial chromosome (BAC) as the transgenic vector. The open reading frame of *Sox2*, which is contained within a single exon, was replaced by the coding sequence for an inducible form of Cre recombinase, CreER™ [17] (Figure 1A). The long sequences flanking the coding sequence in the BAC transgenic construct likely includes most or all regulatory elements to drive the expression of CreER in *Sox2*-expressing cells. Three transgene positive founder mice, numbers 20, 34, and 36, were identified by PCR and confirmed by Southern blot analysis (Figure 1B).

**Figure 2:**
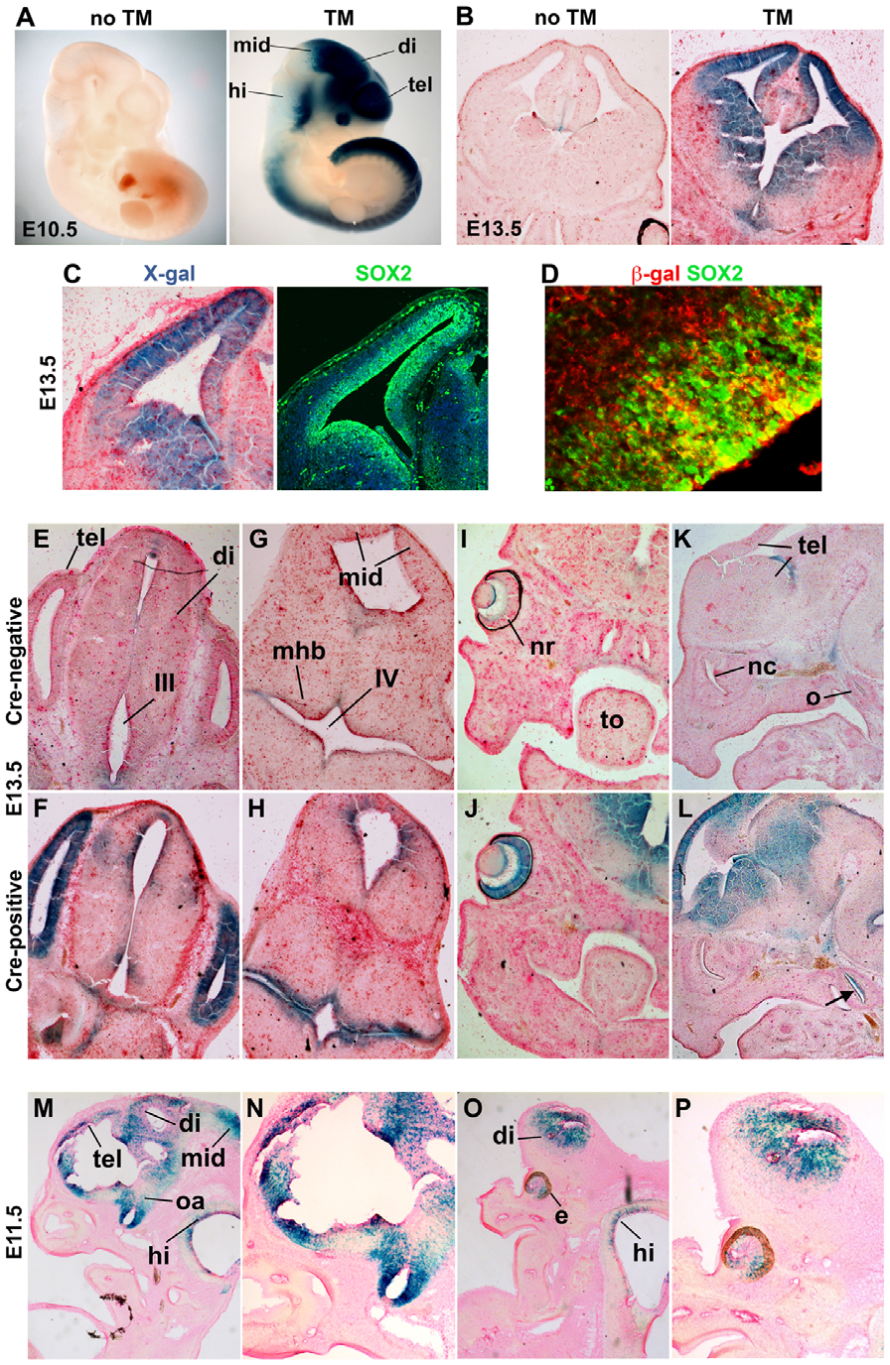
The pattern of CreER activity matches the *Sox2* expression pattern at embryonic stages. Pregnant females were treated with TM (5 mg/35 g body weight) one day before embryos were collected and stained with X-gal or used for immunohistochemistry. Embryos carry the Sox2-CreER BAC transgene (except E,G,I,K) and a Rosa26^lox-stop-lox-lacZ^ reporter allele. Control embryos in (A,B) were from untreated females. (A) At E10.5, β-gal activity is strong in most regions of the CNS except the posterior midbrain and the dorsal hindbrain. There is no detectable β-gal activity without TM. tel, telencephalon; di, diencephalon; mid, midbrain; hi, hindbrain. (B) At E13.5, β-gal activity in the telencephalon is mostly confined to the proliferating stem/progenitor cells in the VZ/SVZ. (C,D) Coronal sections of the telencephalon were used for X-gal and immunoflourescence staining. The areas of X-gal (C) or β-gal (D) staining match the areas of SOX2+ stem/progenitor cells in the VZ/SVZ. Note that the β-gal+ domain is extends beyond the SOX2+ domain, which is likely due to the permanent expression of lacZ in differentiating neurons derived from recombined progenitors from a day earlier when TM was administered. (D) Nuclear SOX2 staining is surrounded by cytoplasmic β-gal staining in the cortical VZ. (E–P) Embryos exposed to TM that either carry the *Sox2-CreER* BAC transgene (F,H,J,L–P) or littermates that do not carry it (E,G,I,K). (E–H) At E13.5, β-gal activity is obvious in the stem/progenitor cells surrounding the lumen of the ventricles in the diencephalon and mid-hindbrain boundary region. (I–L) Strong β-gal activity is also found in the retina and the otic capsule. (M–P) In saggital sections of E11.5 embryos, β-gal activity is strong in the stem/progenitor cells surrounding the ventricles along the anteroposterior axis of the CNS. The supraoptic area and the developing eyes exhibit strong β-gal activity. tel, telencephalon; di, diencephalon; III, third ventricle; IV, fourth ventricle; mid, midbrain; mhb, mid-hindbrain boundary; nr, neural retina; to, tongue; nc, nasal cavity; o, otic capsule; hi, hindbrain; soa, supraoptic area; e, eye.

To analyze the expression pattern of the transgene, the transgenic *Sox2-CreER* mice were crossed to *Rosa26^lox-stop-lox-lacZ^* reporter mice [15]. E10.5 progeny from all three lines exhibited *lacZ* expression specifically in *Sox2*-expressing cells only after TM treatment, which was administered at E9.5 (data not shown). However line number 20 exhibited the strongest *lacZ* expression and was therefore used for further characterization. We found very little or no spontaneous recombination without TM treatment embryonically. At E10.5, one day after TM treatment at E9.5, X-gal staining was intense throughout the neuroepithelium along the anterior-posterior axis with weaker staining in the posterior midbrain and dorsal hindbrain (Figure 2A), mimicking the endogenous *Sox2* expression pattern at these stages [3], [5]. In the developing telencephalon at E13.5, one day after TM treatment, lacZ expression was strong in the proliferating progenitor cells of the ventricular zone (VZ) and subventricular zone (SVZ), but reduced in the outer layers ventrally, where differentiating cells are located (Figure 2B,C).

Anti-SOX2 immunostaining confirmed the expression of *Sox2* in stem/progenitor cells of the VZ/SVZ (Figure 2C,D). Note that the domain of *lacZ* expression extends beyond the proliferative SOX2+ layers both dorsally and ventrally (Figure 2C,D), due likely to the continued expression of *lacZ* in the progeny of the orininally recombined progenitor cells present one day earlier upon TM treatment. Immunofluorescence also indicates that cytoplasmic β-gal and nuclear SOX2 co-label stem/progenitor cells in the telencephalon (Figure 2D). In the diencephalon and the mid and hindbrain boundary region, X-gal staining is weaker but clearly also present in some stem/progenitor cells surrounding the ventricles (Figure 2E–H). In addition, strong lacZ expression was found in the developing neural retina and otic capsule (Figure 2I–L), consistent with important roles for *Sox2* in eye and ear development [18], [19]. X-gal staining of E11.5 sections revealed similar results as for E13.5 brain regions. In addition to stem/progenitor cells around the ventricles, the supraoptic area exhibited strong *lacZ* expression (Figure 2M–P). Outside the nervous systems, little or no X-gal staining was observed at E11.5.

Overall, the TM induced Cre activity accurately recapitulates aspects of the endogenous *Sox2* expression pattern previously determined using *Sox2-*β*geo* and *Sox2-GFP* knock-in mice, in which β-gal activity or GFP signal was present along the entire anterior-posterior axis of the developing nervous system [3], [6]. The targeting of Cre recombinase to *Sox2*-expressing cells during development in this transgenic mouse line indicates that it can be used to manipulate genes of interest in *Sox2*-expressing stem/progenitor cells in the CNS, particularly in the forebrain and the spinal cord, in a temporally specific manner during development.

### The expression of *Sox2-CreER* in adult neurogenic regions

During adulthood, new neurons are continuously generated in two brain regions, the anterior subventricular zone (SVZ) and the subgranular zone (SGZ) of the hippocampal dentate gyrus. In the SVZ, the slow-dividing GFAP+ radial-glia like cells exhibit characteristic stem cell features including self-renewal and pluripotency. These stem cells are thought to divide asymmetrically to produce a new stem cell and a fast-proliferating progenitor cell that in turn generates neuroblasts which migrate along the rostral migratory stream (RMS) to reach the olfactory bulb and differentiate into interneurons. In the SGZ, similar GFAP+ stem cells give rise to progenitor cells that generate neurons migrating short distances radially to populate the granule cell layer [20]. Previously, *Sox2* expression was observed in these adult neurogenic regions. For instance, an anti-SOX2 antibody labels proliferating precursor cells (marked by incorporation of BrdU) and GFAP+ cells that are thought to be neural stem cells [6]. However, since GFAP also labels mature astrocytes, the GFAP+/SOX2+ cells could potentially represent astrocytes instead of stem cells in the neurogenic regions. In addition, although *Sox2*-expressing cells derived from the SVZ and SGZ can form neurospheres when grown in culture [3], [11], transit amplifying progenitor cells can also form neurospheres and behave as stem cells in culture [21]. Thus, whether *Sox2* is expressed in self-maintaining stem cells, transit amplifying cells, and/or astrocytes in vivo remains uncertain.

**Figure 3:**
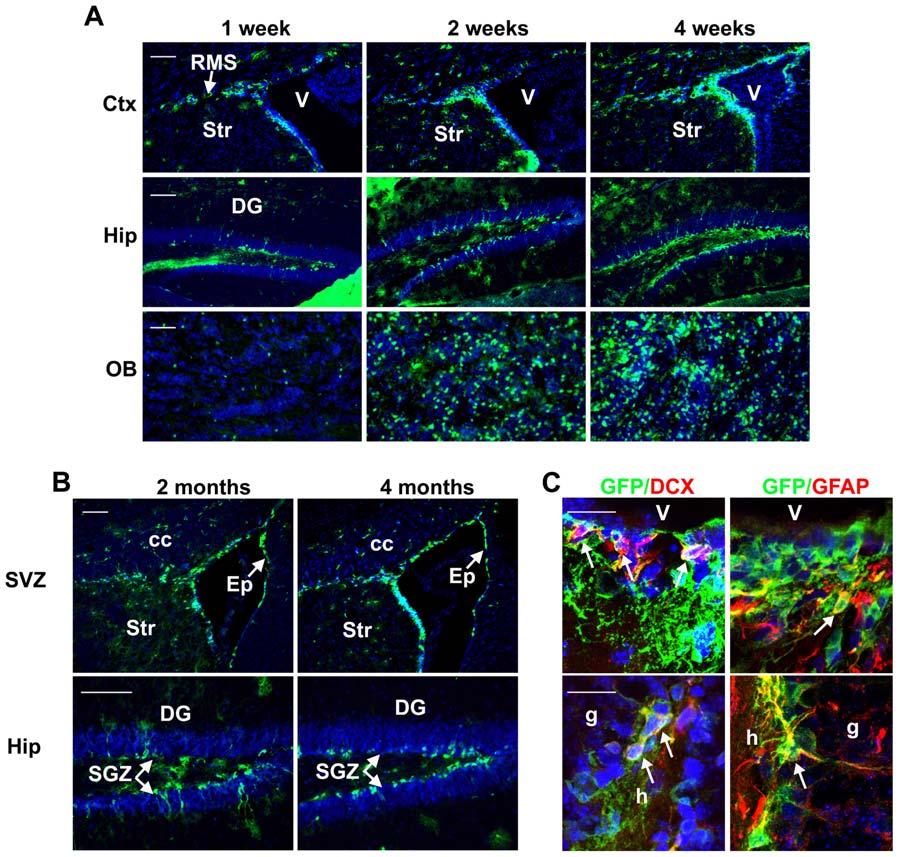
Sox2-CreER activity is observed in the adult neurogenic regions. Two to three month old Sox2-CreER;RCE mice were treated with TM and brains were analyzed at different time points after treatment. (A) Representative pictures of the SVZ, dentate gyrus of the hippocampus, and olfactory bulb at the time points indicated. Note that the number of GFP+ cells in the SVZ, RMS, dentate gyrus, and olfactory bulb increased over time from 1 to 4 weeks after TM treatment. Scale bars: 100 µm. (B). Representative pictures of the SVZ and dentate gyrus at 2 months and 4 months after TM treatment. The presence of a large number of GFP+ cells in the SVZ, RMS, and SGZ indicates that the *Sox2*-expressing cells that were initially recombined continue to produce new neurons over a long period of time. Scale bars: 100 µm. (C) Representative confocal images of sections immunostained for GFP and either the radial glia-like stem cell marker GFAP or the neuroblast marker DCX at 4 months after TM treatment. The presence of GFAP+GFP+ cells and DCX+GFP+ cells in the SVZ and SGZ at 4 months after TM treatment (arrows) confirms the self-renewal and long-term neurogenic ability of initially labeled *Sox2*-expressing cells and functionally defines these cells as stem cells. Scale bars: 20 **µ**m. SVZ, subventricular zone; DG, dentate gyrus; V, ventricle; Str, striatum; RMS, rostral migratory stream; Ctx, neocortex; Hip, hippocampus; OB, olfactory bulb; CC, corpus callosum; SGZ, subgranular zone; Ep, ependyma; h, hilus; g, granular layer.

To examine whether *Sox2*-expressing cells in the adult neurogenic regions include neural stem cells, we used our *Sox2-CreER* transgenic mice crossed with a conditionally activated GFP reporter mouse, RCE [16], to trace the long term fate of these cells. In the *Sox2-CreER;RCE* mice, Cre-recombined cells, which should include *Sox2*-expressing cells as well as all their progeny, will be labeled with green fluorescence protein (GFP). In 2–3 month old mice, we observed very few GFP+ cells in the absence of TM, indicating little or no leaky recombination. With TM treatment, we observed a gradual increase in the number of GFP+ cells in the SVZ, RMS, olfactory bulb, SGZ, and the granule cell layer of the dentate gyrus from 1 week to 1 month after TM treatment (Figure 3A), as would be expected if *Sox2*-expressing cells are involved in adult neurogenesis. Consistent with previous *Sox2* expression studies [6], non-dividing ependymal cells lining the lateral ventricle also become GFP+ (Figure 3B). To examine whether *Sox2* is indeed expressed in neural stem cells, which unlike transient amplifying progenitors or GFAP+ astrocytes can self-renew and support long term neurogenesis, neurogenic regions were examined 2–4 months after TM treatment. Previously, lineage tracing of progenitor cells using *Mash1-CreER* demonstrated that labeled cells in the SVZ and RMS disappeared within 1 month of TM treatment due to the lack of progenitor cell self-renewal [22]. In contrast, in *Sox2-CreER;RCE* mice, the GFP+ cells remained abundant in the SVZ, RMS, and SGZ up to 4 months after TM treatment (Figure 3B). In particular, GFP+/GFAP+ cells with morphologies characteristic of radial glia stem cells (with few long processes) are still detected in the SVZ and SGZ and DCX+/GFP+ neuroblasts are still present in the SVZ, RMS, and SGZ in the dentate gyrus (Figure 3C and data not shown). These data provide strong evidence that *Sox2*-expressing cells include neural stem cells that are capable of self-renewal and long-term neurogenesis in the SVZ and SGZ. Therefore, *Sox2* can serve as an additional adult neural stem cell marker to isolate and study neural stem cells. Moreover, *Sox2-CreER* mice will be a useful tool to target genes in adult neural stem cells or to trace their behaviors under different experimental conditions.

### *Sox2-CreER* expression in non-neurogenic brain regions in the adult

**Figure 4:**
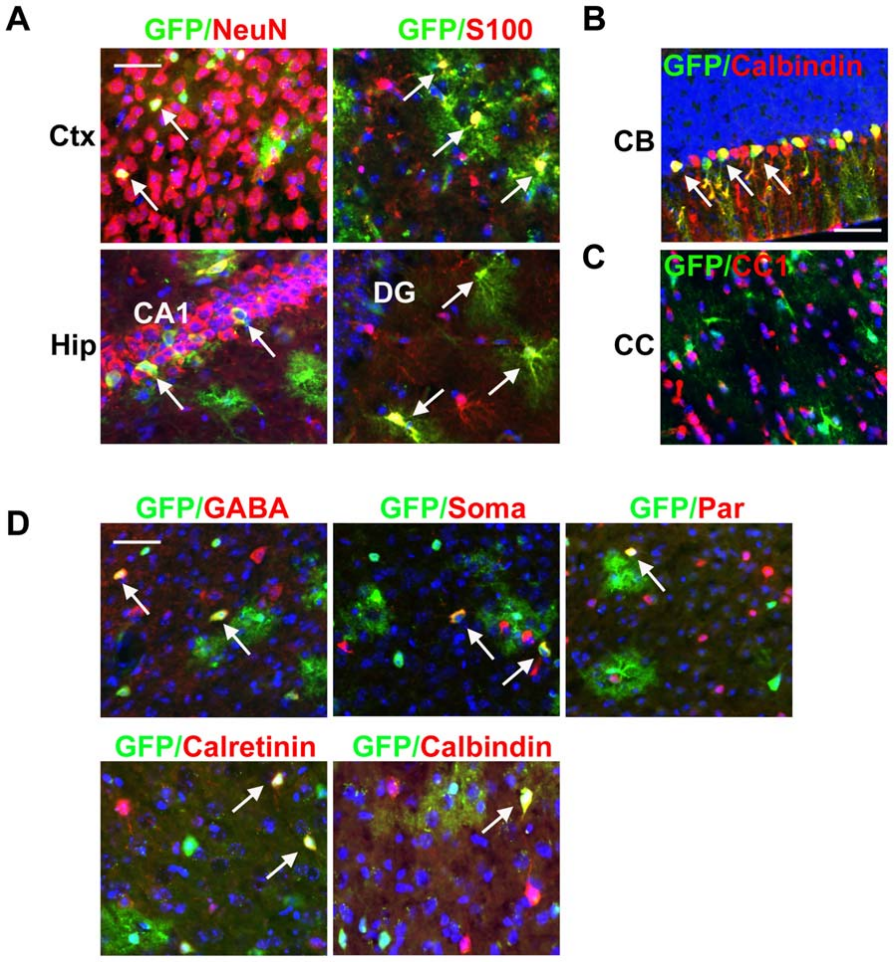
The pattern of CreER activity suggests that *Sox2* is also expressed in differentiated cells. Two to three month old Sox2-CreER;RCE mice were treated with TM and brains were analyzed 2 or 4 weeks after treatment. (A) In the neocortex and hippocampus, some GFP+ cells express the neuronal marker NeuN or the astrocyte marker S100 (arrows). (B) In the cerebellum, GFP+ cells co-label with the Purkinje cell marker calbindin (arrows). (C) In the corpus collosum, GFP+ cells are not co-labeled with the oligodendrocyte marker CC1. (D) Some cortical GFP+ cells are co-labeled with the interneuron marker GABA (arrows). Moreover, GFP staining co-labeled with markers for different subtypes of interneurons (arrows). CC: corpus collosum, CB: cerebellum, DG: dentate gyrus, Soma: somatostatin, Par: parvalbumin. Scale bars correspond to 40 µm.

A previous study detected sparse neuron-like cells stained with an anti-SOX2 antibody in the mature cortex and other differentiated regions [6]. Moreover, *Sox2* hypomorphic mice exhibit neuronal degeneration in several adult brain regions, which may be associated with neurological disorders including epileptic spikes and motor dysfunction [6]. In humans, *SOX2* mutations can also result in neurological disorders including cognitive defects and seizures [23], [24], [25]. These observations suggest that *Sox2* is expressed in differentiated cells and that it is required for normal neuronal functions. In the *Sox2-CreER;RCE* mice, in addition to GFP+ cells in the neurogenic regions, we also observed GFP+ cells in the cortex, hippocampus, basal ganglia, thalamus, hypothalamus, midbrain, hindbrain, and cerebellum (Figure 4 and data not shown).

In this study, we mainly focused on the neocortex and hippocampus. In ∼3 month old adult mice, GFP+ cells with both neuronal morphologies (large cell bodies and dendritic-like projections) and astrocytic morphologies (relatively small cell bodies with fine bush-like processes) were observed at 2 weeks, 4 weeks, and 4 months after TM treatment. Co-labeling with the neuronal marker NeuN and astrocytic marker S100, but not the oligodendrocyte marker CC1 in either the corpus callosum or neocortex, was detected 3–4 weeks after TM treatment, confirming that Sox2-CreER is expressed in mature neurons and astrocytes (Figure 4A,C and data not shown). In the neocortex, GFP+ cells are detected in all 6 layers from anterior to posterior areas. In the hippocampus, outside the dentate gyrus, GFP+ neurons are mainly detected in the CA1 region (data not shown). Consistent with previous immunohistochemical analyses [12], a majority of S100+ astrocytes in the hippocampus appear GFP+. Furthermore, using markers to label different neuronal subtypes, we found that in addition to neurons with pyramidal neuron morphology, GFP+ neurons in the neocortex can be GABA+ interneurons, including calbindin+, parvalbumin+, somatostatin+, and calretinin+ subtypes (Figure 4D). GFP+ neurons and astrocytes are also detected in other brain areas including the basal ganglia, amygdala, thalamus, hypothalamus, midbrain regions, hindbrain regions, and cerebellum where GFP+/Calbindin+ Purkinje cells are detected specifically in lobule IV–X (Figure 4B).

Previously, using *Sox2-GFP* knock-in mice, GFP expression was not detected in mature NeuN+ neurons [11]. This discrepancy with our results may be due to the relatively weak expression of *Sox2* in mature neurons, which is consistent with the weak staining observed using an anti-SOX2 antibody [6], and to the fact that in the *Sox2-GFP* knock-in mice GFP expression is driven by the relatively weak *Sox2* promoter, whereas in our study once recombination has occurred GFP expression is under the control of a strong CAG promoter at the *Rosa26* locus. The GFP+ subpopulations of neurons and astrocytes that express *Sox2-CreER* observed in this study may underlie previously uncharacterized functional heterogeneity in these cell types and could potentially provide insight into *SOX2*-associated neurological disorders.

### *Sox2-CreER* activity outside of the nervous system in the adult

**Figure 5:**
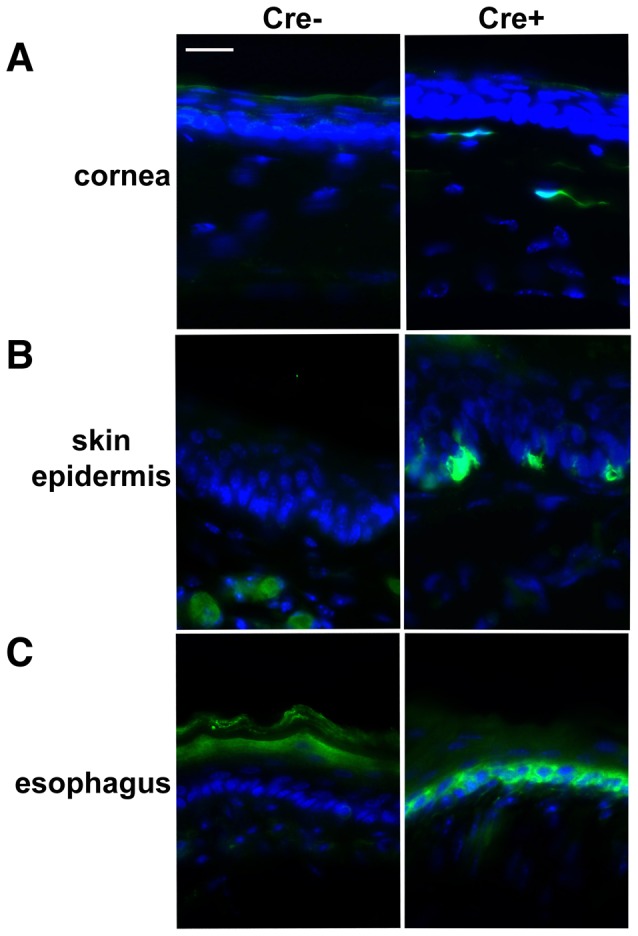
Sox2-CreER activity is detected outside the nervous system in adults. Immunostaining for GFP in different tissues of 6 months old wild type and Sox2-CreER;RCE mice 5 weeks after TM treatment. GFP staining is found in keratocytes of the cornea (A), basal cells of the skin epidermis (B), and basal cells of the esophageal epithelium (C).

Previously, using *Sox2-GFP* knock-in mice, *Sox2* expression was detected in multiple adult tissues, including epithelial layers of the lens, trachea, lungs, esophagus, dermal papillae of the hair follicles, and gastric units of the glandular stomach [26]. For comparison sake, we examined the expression pattern of *Sox2-CreER* in our BAC transgenic line. Five weeks after TM treatment of adult Sox2-CreER;RCE mice basal cells of the esophageal epithelium are GFP+ (Figure 5C), consistent with the previous study. Scattered GFP+ basal cells in the skin epidermis, which was shown to express *Sox2* previously by immunohistochemistry [26], were also detected (Figure 5B). However, no GFP+ cells were found in the epithelia of the lens, trachea, and lung, nor in the dermal papillae of the hair follicles or the glandular stomach (data not shown). Unexpectedly, we found GFP+ keratocytes in the cornea, which have not previously been linked to *Sox2* expression (Figure 5A). The lack of *Sox2-CreER* expression or activity in certain tissues in the BAC transgenic mice that were previously found to express *Sox2-GFP* in knock-in mice may due to weaker expression or less efficient recombination in those tissues. The possibility that some *Sox2* transcriptional regulatory elements are missing in the BAC construct can also not be excluded. Nevertheless, the *Sox2-CreER* mice will be useful to target genes in the skin and esophageal epithelia as well as neural stem cells in the adult.
